# Detecting Careless Responding in Survey Data Using Stochastic Gradient Boosting

**DOI:** 10.1177/00131644211004708

**Published:** 2021-04-19

**Authors:** Ulrich Schroeders, Christoph Schmidt, Timo Gnambs

**Affiliations:** 1 University of Kassel, Kassel, Germany; 2 eoda GmbH, Kassel, Germany; 3 Leibniz Institute for Educational Trajectories, Bamberg, Germany

**Keywords:** careless responding, gradient boosted trees, data cleaning, response times, outlier detection

## Abstract

Careless responding is a bias in survey responses that disregards the actual item content, constituting a threat to the factor structure, reliability, and validity of psychological measurements. Different approaches have been proposed to detect aberrant responses such as probing questions that directly assess test-taking behavior (e.g., bogus items), auxiliary or paradata (e.g., response times), or data-driven statistical techniques (e.g., Mahalanobis distance). In the present study, gradient boosted trees, a state-of-the-art machine learning technique, are introduced to identify careless respondents. The performance of the approach was compared with established techniques previously described in the literature (e.g., statistical outlier methods, consistency analyses, and response pattern functions) using simulated data and empirical data from a web-based study, in which diligent versus careless response behavior was experimentally induced. In the simulation study, gradient boosting machines outperformed traditional detection mechanisms in flagging aberrant responses. However, this advantage did not transfer to the empirical study. In terms of precision, the results of both traditional and the novel detection mechanisms were unsatisfactory, although the latter incorporated response times as additional information. The comparison between the results of the simulation and the online study showed that responses in real-world settings seem to be much more erratic than can be expected from the simulation studies. We critically discuss the generalizability of currently available detection methods and provide an outlook on future research on the detection of aberrant response patterns in survey research.

Deliberate misreporting is a serious threat to the reliability and validity of social science research. Increasing evidence suggests that some participants engage in dishonest behavior and cheat in unproctored achievement tests (Steger, Schroeders, & Gnambs, 2020), distort their responses in line with prevalent social norms (Gnambs & Kaspar, 2015), or simply do not invest the necessary effort to properly process a survey question and indicate an elaborated response (Huang et al., 2015; Litman & Robinson, 2020). Rather, a substantial proportion of survey respondents adopt cognitive shortcuts, for example, by using specific response patterns such as selecting the same response category for multiple items instead of evaluating the actual item content (Johnson, 2005). Particularly, in web-based studies, careless responses have become a major hindrance to data quality (Bowling & Huang, 2018; Weiner & Dalessio, 2006), which can substantially affect study results (Arias et al., 2020; Huang et al., 2015). Accordingly, various data screening methods have been proposed to identify careless respondents (Meade & Craig, 2012; Niessen et al., 2016), such as probing items that directly assess test-taking behavior (e.g., bogus items), auxiliary or paradata (e.g., response times), or data-driven techniques (e.g., Mahalanobis distance). In contrast, prediction methods that are commonly used in the machine learning literature, but (as of yet) are rarely used in sociological and psychological research, have been somewhat neglected. Thus, we introduce stochastic gradient boosting (Friedman, 2001) as a means to identify careless respondents in self-report surveys. The novel approach is compared with established techniques using simulated data that exhibit a clear allocation of cases to regular and careless respondents. In addition, empirical data from a web-based experiment in which participants were instructed to display different types of test-taking behavior (regular, inattentive) probe the usefulness of the machine learning algorithm as compared with traditional techniques for the detection of careless respondents.

## Types and Sources of Careless Responding

The phenomenon of *careless responding* has been explained with different nuances and labels: “content nonresponsivity” (Nichols et al., 1989, p. 239), “careless inattentiveness” (Johnson, 2005, p. 104), “insufficient effort responding” (Huang et al., 2012, p. 100), or “careless/insufficient effort” (Arias et al., 2020). Most commonly, it is considered a bias in survey responses disregarding the actual item content. Sometimes, this has been described as random responding (e.g., Berry et al., 1992). However, this term is misleading because these responses can also follow some nonrandom pattern (e.g., a recurring sequence of 1-2-3-4-5) and, more important, humans are incapable of producing true random sequences (Figurska et al., 2008). But the distinction between careless and random responses may point to the difference between intentional and nonintentional response behavior. As Niessen et al. (2016) reflect on a reviewer’s comment, careless responding is perceived as “the more benign form of aberrant responding . . . , whereas random responding is more blatant and intentional” (p. 1). Accordingly, in our understanding, careless responding covers both intentionally and unintentionally answering items (Meade et al., 2017, p. 417) without the intention of creating a specific image of oneself. Thus, careless responding does not include, for example, malingering—the fabrication, feigning, or exaggeration of physical or psychological symptoms to achieve some beneficial outcome (e.g., early retirement, mitigating punishment, claim for compensation)—which is a common concern in clinical and forensic psychology (Mittenberg et al., 2002). This distinction between careless responding and faking or cheating may be subtle, but it is important because the latter hinges on participants’ ability to fake (Geiger et al., 2018) and is presumably much harder to detect (e.g., Karabatsos, 2003).

Prevalence estimates on the extent of careless responding vary considerably, from modest 3.5% (Johnson, 2005), over 3% to 9% (Maniaci & Rogge, 2014), 10.6% (Kurtz & Parrish, 2001) and 10% to 12% (Meade & Craig, 2012) up to 35% to 46% (Oppenheimer et al., 2009). The large heterogeneity in estimates is presumably due to the different operationalizations of *true* careless responding. Moreover, it is reasonable to assume that studies differ in their extent of careless responding depending on the study’s intention, the recruitment procedure, and incentives given. Regarding the consequences, careless responding introduces systematic bias, whereas random responses add noise to the measurement. Ultimately, such error variances reduce reliability estimates, measurement precision, and also attenuate or inflate correlations and, therefore, pose a threat to validity (Hong et al., 2020; Huang et al., 2015; McGrath et al., 2010). In simulation studies, Credé (2010) convincingly showed that base rates of careless responding as low as 5% can affect correlations in a similar manner as, for example, range restriction or score unreliability. Accordingly, variance–covariance-based methods are also affected by careless responding. For instance, in case of scales with partially reversely coded items, careless responding might contribute to the emergence of an additional method factor capturing the variance of some negatively worded items of the same scale. Already 10% to 20% of careless respondents in the sample are sufficient to render a single-factor confirmatory model incorrect (Woods, 2006; see also Huang et al., 2012). In the same vein, Gnambs and Schroeders (2020) compared different measurement models for the factor structure of the *Rosenberg Self-Esteem Scale* across several continuous moderators and concluded that the scale becomes more unidimensional with increasing reading competence. Thus, superficial reading or comprehension problems are an obvious source of careless responding, but there are several, potential reasons (e.g., test-taking motivation).

## Traditional Techniques for the Detection of Careless Respondents

Given the risk of biased results, a number of different methods have been proposed to detect careless respondents. A rough distinction can be made between (a) special items or scales assessing participants’ care, (b) the evaluation of auxiliary data from computerized testing, and (c) statistical, data-driven methods. The first group includes *bogus items* with an obvious correct answer (e.g., “I am paid biweekly by leprechauns”), or *instructed response items* to which participants have to react correctly (e.g., “Please click strongly agree”; Gummer et al., 2018, or the so-called blue dot task; Oppenheimer et al., 2009), or self-report measures often placed at the end of a survey that directly ask for participants’ diligence and engagement (e.g., “I put forth my best effort in responding to this survey”; Meade & Craig, 2012). However, the usefulness of such items is still debated (Curran & Hauser, 2019), because their inclusion can result in negative spillover effects by irritating participants or introducing reactance.

The second group of indices relies on so-called paradata to gain further insights into participants’ test-taking behavior (Couper, 2005). Paradata are additionally recorded in computerized testing and include, among others, log data (Kroehne & Goldhammer, 2018), response latencies (Leiner, 2019), keystrokes, and mouse clicks (Kieslich & Henninger, 2017; Olson & Parkhurst, 2013). Major advantages of paradata are that they can unobtrusively be assessed and are hard to fake. For example, click counts and page timing have been used to flag bot-generated answers in a web-based survey (Buchanan & Scofield, 2018). In the same vein, Steger, Schroeders, and Wilhelm (2020) demonstrated in an experimental setting, comparing a proctored with an unproctored knowledge assessment, the superiority of paradata such as response times and focus shift of browser tabs in comparison to classical self-report scales (e.g., honesty scales) in detecting cheaters. And recently, both responses and auxiliary response time information was used in a mixture hierarchical modeling approach to detect aberrant responses (Wang et al., 2018; Wang & Xu, 2015).

Finally, several data-driven techniques, such as statistical outlier methods, consistency analysis, or response pattern functions (see Table 1), are conducted after the assessment to identify careless respondents. The detection and removal of *statistical outliers* (i.e., data points that differs significantly from other observations) is one of the most common methods of data cleaning. Multivariate approaches such as the Mahalanobis distance evaluate the entire response pattern in a series of items (e.g., a scale) to identify respondents with aberrant response behavior. Previous research indicated high sensitivity and specificity of outlier detection for complete random responses, but not for the midpoint response style or more sophisticated cheating strategies (Maniaci & Rogge, 2014; Meade et al., 2017). *Consistency analysis* comprises a heterogeneous set of techniques (Curran, 2016; Maniaci & Rogge, 2014; Meade & Craig, 2012), all relying on the basic notion that careless respondents produce responses that are internally inconsistent. For example, the odd–even consistency is calculated by breaking each individual’s responses into even–odd items sets for each unidimensional subscales. On the item level, the within-person correlations between highly correlated item pairs (irrespective of the content) are called psychometric synonym/antonym scores. Although such consistency indices yield good, sensitive detection of careless respondents (Curran, 2016), they are accompanied by a variety of difficulties, for example, the necessity of relatively homogeneous, redundant scales with similarly (or oppositely) formulated items. Last, *response pattern functions* are used to detect uncommon patterns in parts or the complete response vector of an individual in comparison to others.^1^ Some of them are easy to calculate such as the longstring index (i.e., the number of consecutive items answered with the same response alternative; Johnson, 2005) or the inter-item standard deviation, which is also known as intraindividual response variability (IRV; i.e., standard deviation across a response vector; Marjanovic et al., 2015). They are useful if the items can be assigned to different constructs and some of them are negatively worded. More complex procedures include the number of Guttman errors (Curran, 2016) or person-fit statistics (Meijer, 1996), which have been devised for achievement data with a dichotomous answer format, whereas there are only few procedures for polytomous items (Emons, 2008; Tendeiro et al., 2016), especially in a multidimensional context (Drasgow et al., 1985; Glas & Dagohoy, 2007). More specifically, person-fit statistics have been used to identify participants with spuriously low or high test scores by comparing participants’ actual responses with the expected responses (Karabatsos, 2003) and have been reported to detect both deliberate cheating (e.g., answer copying; Sotaridona & Meijer, 2002) and random responding (Niessen et al., 2016).

**Table 1.** Overview of Data-Driven Mechanisms to Detect Careless Respondents.

Index (Abbr.)	Description	Strengths	Weaknesses	Key references
Statistical outlier functions				
Mahalanobis distance (Maha.)	Multivariate distance between a respondent’s response vector and the vector of sample means	• Easy to calculate and understand • Widespread usage	• Effective only for truly random responses	Mahalanobis (1936)*, Meade & Craig (2012)
Consistency analysis				
Synonym/antonym score (Ant.)	Within-person correlation between highly correlated item pairs (e.g., |*r*| > .60)	• Good, sensitive detection of careless respondents	• For semantic synonym/antonym score similar/contrasting worded items • Varying and arbitrary cutoff for correlation	Meade & Craig (2012), Maniaci & Rogge (2014), Curran (2016)
Even–odd consistency (EvenOdd)	Within-person correlation across unidimensional subscales formed by even–odd split halves	• High scale dependency	• Relies on unidimensional scales • Sufficient amount of items	Meade & Craig (2012), Curran (2016)
Intraindividual response variability (IRV)	Intraindividual standard deviation across a set of consecutive item responses	• Very easy to calculate and understand	• Should be calculated across multiple constructs and reversely coded items • Both low and high variability might indicate careless responding	Marjanovic et al. (2015)*, Dunn et al. (2018)
Response pattern functions				
Longstring (Long.)	Maximum (or average) of consecutive items answered with the same response option	• Easy to calculate and understand	• Uniform response scale • Needs larger sets of items covering different constructs • Arbitrary cutoff	Johnson (2005)*
Number of Guttman errors	Number of item pairs that behave contrary to expectations regarding the solution probabilities	• Nonparametric version	• Relies on large sample sizes • Rather complicated to calculate	Meijer et al. (1994)*, Niessen et al. (2016)
Polytomous *U*_3_ person-fit statistics	Extent to which a person's nonparametric polytomous IRT estimate matches the probability of correctly solving items	• Nonparametric version	• Relies on large sample sizes • Rather complicated to calculate • Presupposes unidimensional scales	Emons (2008)*
*Z*_h_ statistics (*Z*_h_)	Extent to which a person’s polytomous IRT estimate corresponds to the probability of correctly solving items	• Uses information on the structure of the meausre	• Relies on large sample sizes • Rather complicated to calculate	Drasgow et al. (1985)*

*Note*. References marked with an asterisk (*) are key references. The intraindividual response variability is also known as inter-item standard deviation and can also be classified as a response pattern function. The abbreviation in parentheses is used in the Results section. IRT = item response theory.

## Stochastic Gradient Boosted Trees

Although machine learning methods have existed in computer science for decades, they have only recently found wider application in psychology for the analysis of large and complex data structures (Fan et al., 2014), including the prediction of personality from online social networking behavior (Kosinski et al., 2013) or the prediction of physiological health (e.g., diabetes, hypertension) through personality nuances (i.e., items; Seeboth & Mõttus, 2018). In psychological assessment, Zopluoglu (2019) used an extreme gradient boosting algorithm, which is an efficient implementation of gradient boosting (Friedman, 2002), to identify test-takers with preknowledge more precisely compared with more traditional item exposure measures (area under the receiver operating characteristic curve [AUROC] = .930). Thus, the algorithm might help in classification problems such as separating careless from diligent respondents in case the class labels are known. Besides the good predictive performance in similar contexts (Zopluoglu, 2019), also the properties of the algorithm favor this method over others (see Friedman, 2002).

**Figure 1.**
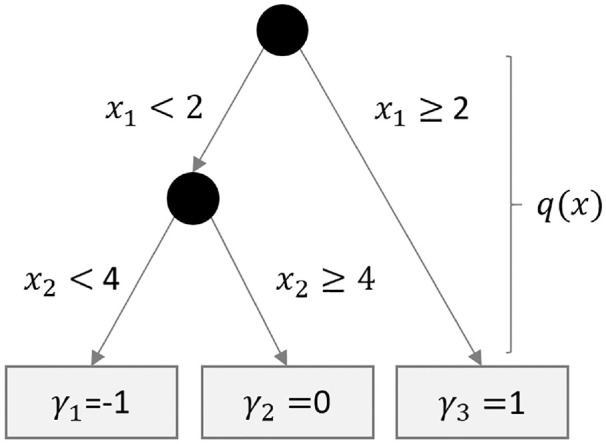
Example of a decision tree with two nodes and three leaves.

In general, decision trees are a versatile and increasingly popular method for the analysis of complex data structures with many variables and nonlinear relationships that do not require the a priori specification of a functional form between an outcome and its predictors (James et al., 2017). They sequentially partition the sample into mutually exclusive classes and, thus, build a tree-like structure such as in Figure 1. Let *y_i_* denote the outcome for respondent i∈{1,...,I} and $x_{i}=\left\{x_{i1},x_{i2},\ldots ,x_{\mathrm{iq}}\right\}$ a *Q*-dimensional vector of predictors. Then, a set of decision rules and associated parameter values is identified that splits the data at each branch of the tree into two smaller subsets according to a variable in *x*, thus, resulting in a tree that consists of *J* end points (i.e., leaves). Given a tree structure *T* with its decision rules q(x) that maps each observation *i* to one of the *k* leaves and a vector of scores on each leaf $\gamma =\left\{\gamma_{1},\gamma_{2},\ldots ,\gamma_{k}\right\}$ the regression relationship between *y_i_* and *x_i_* can be formally expressed as

(1) $${\hat{y}}_{i}=f\left(x_{i};T\right),$$

with *f* as a function describing the tree. The unknown tree parameters *T* are obtained by minimizing a differentiable loss function *L* that measures the goodness of the prediction for each data point:

(2) $${\hat{T}}_{m}=\arg\min\sum_{i=1}^{N}L\left(y_{i},{\hat{y}}_{i}\right).$$

As the present study uses a binary outcome that represents the regular versus careless respondents group, a logistic loss function *L* is used

(3) $$L\left(y_{i},{\hat{y}}_{i}\right)=y_{i}\ln\left(1+e^{-{\hat{y}}_{i}}\right)+\left(1-y_{i}\right)\ln\left(1+e^{{\hat{y}}_{i}}\right).$$

Single trees usually have a rather poor predictive performance, but can be combined as ensembles for higher accuracies (Breiman, 2001). The gradient boosting machine (GBM; Friedman, 2001) sequentially estimates *M* decision trees by choosing the optimal split points for *x_i_*. At the same time, the maximum number of leaves and splits is limited to guarantee that each tree acts as a weak learner and does not dominate the prediction. The *M* decision trees are added one at a time using a gradient descent procedure. The loss function *L* is approximated by calculating its gradient with respect to the predicted value of the ensemble and adding a new tree to the ensemble, if the tree moves the loss in the direction of the prediction values. Following this logic, the prediction $\hat{y}$ at iteration *m* can be expressed as the prediction at the previous iteration ${\hat{y}}_{i}^{\left(m-1\right)}$ and the prediction by the current tree $f_{m}\left(x_{i};T_{m}\right)$ , thus rewriting the loss function at iteration *m* as

(4) $${\hat{T}}_{m}=\arg\min\sum_{i=1}^{N}L\left(y_{i},{\hat{y}}_{i}^{\left(m-1\right)}+f_{m}\left(x_{i};T_{m}\right)\right).$$

The numerical optimization algorithm suggested by Friedman (2001) reformulates Equation (4) using the negative gradient *g_im_* for the *i*th observation at iteration *m* and proceeds in three steps. First, *g_im_* is estimated for each observation *i* as

(5) $$g_{\mathrm{im}}=-\left[\frac{\partial L(y_{i};f_{\left(m-1\right)}\left(x_{i};T_{\left(m-1\right)}\right)}{\partial f\left(x_{i};T_{\left(m-1\right)}\right)}\right].$$

These pseudo-residuals reflect the potential reduction in loss and, in the second step, are used as outcomes for estimating the parameters for the decision tree at iteration *m* using Equation (5). Finally, the predictions are updated to derive the predicted values at iteration *m* as

(6) $${\hat{y}}_{i}^{m}={\hat{y}}_{i}^{\left(m-1\right)}+\lambda \cdot f_{m}\left(x_{i};{\hat{T}}_{m}\right),$$

with  λ as the gradient descent step size (i.e., learning or shrinkage parameter) that controls the rate at which the algorithm updates the predictions. Friedman (2002) showed that incorporating a boosting approach in the algorithm substantially improves the predictive performance. Thus, stochastic gradient boosted trees do not use the entire sample in Step 2 but are limited to a random subsample (without replacements) of observations.

In stochastic gradient boosting, various tuning parameters need to be specified that affect the performance of the algorithm. Because there is no convergence criterion, the number of iterations *M* determines the amount of residual error. The maximum interaction depth determines the number of splits allowed in the tree, whereas the leaf size indicates the minimum number of observations in a given leaf. Larger interaction depths and smaller leaves sizes allow for more complex models, but also lead to less stability and higher computational demands (Berk, 2017). In addition, parameters regarding the minimum impurity decreasement for a split to be considered worthy, the learning rate λ, and the number of trees are commonly optimized in grid searches. Small values for the learning rate λ not only slow down the learning, requiring more iterations, but also improve the stability of the predictions.

## The Present Study

The main objective of the study is to evaluate different statistical techniques to identify participants who likely engaged in careless responding. Therefore, we implemented different post hoc statistics that have previously been described in the literature and that serve as a benchmark (e.g., statistical outlier methods, consistency analyses, and response pattern functions, see also Table 1). To identify careless respondents, we also used a GBM (Friedman, 2002). In comparison to other machine learning algorithms, the GBM has several advantages: First, it is a black box approach that does not require the a priori specification of a functional relationship between the predictors and group membership (regular vs. careless respondents), which is advantageous because the relationship is basically unknown. Such a model evaluation favors prediction over explanation (Shmueli, 2010). Second, the GBM can capture complex relationships (James et al., 2017) between the predictors (i.e., interactions) and between predictors and outcome (i.e., nonlinear relations). Third, the GBM is eligible to search for patterns in complex data structures with many heterogeneous variables, including paradata such as response times. Paradata is considered more difficult to counterfeit because the recording is done incidentally and clear assumptions of the test-takers about the relation between paradata and careless responding are missing. The evaluation of binary classifiers (e.g., specificity, sensitivity) is done for traditional detection mechanisms and gradient boosted trees with simulated data and with empirical data of an experimental study.

## Method

### Study 1: Simulation Study

#### Data Generation

In comparison to real data where the prevalence of careless responding is usually unknown (if not experimentally induced or otherwise controlled for), Monte Carlo simulations offer the advantage that different response patterns can be modeled reliably, so that the classification quality of the detection mechanisms can be evaluated without measurement uncertainty. Although they allow nuanced conclusions about the specific procedures, they can be criticized for their idealized distributions of both regular and irregular response patterns. In general, Monte Carlo simulations require a large number of specifications (see also previous simulations by Credé, 2010; Meade & Craig, 2012; Niessen et al., 2016); we describe the most important ones below and refer for detailed specifications to an Open Science Framework (OSF) repository (Soderberg, 2018) in which we provide all data and syntax files to foster transparency and reproducibility: https://osf.io/mct37.

We simulated nine data conditions in a 3 × 3 design manipulating the amount of careless respondents in the overall sample (5%, 10%, 15%) and three types of response styles (random, midpoint, and fixed pattern respondents; see also Meade & Craig, 2012). We aligned the data generation of the simulation study as closely as possible to the empirical data (which will be described as Study 2) to enable a proper comparison between both studies. More precisely, we simulated valid responses to 60 ordinal variables with five response categories based on the empirical correlation matrix and the response distribution from Study 2 (see OSF repository for the syntax) using the R package genOrd (Version 1.4.0; Ferrari & Barbiero, 2012). With regard to the sample size, we also closely mirrored the conditions of the empirical study allowing for a direct comparison of the detection rates with simulated and empirical data (for more information see Appendix A in the OSF repository).

In line with previous simulation studies,^2^ we differentiate three types of careless respondents: (a) random respondents, (b) midpoint respondents, and (c) fixed pattern respondents. In more detail, the answers of the *random respondents* were randomly drawn from a uniform distribution across all response categories (i.e., 20%, 20%, 20%, 20%, 20%). Accordingly, the responses can be considered as completely random, which is not necessarily a realistic condition. The *midpoint response styles* mirrors the response behavior of “persons that may stick primarily to the scale midpoints over concerns of being identified via outlier analysis” (Meade & Craig, 2012, p. 448). In the simulation, the midpoint response style camouflaging inattentive responses is characterized by successively higher probabilities for the middle categories (i.e., 5%, 20%, 50%, 20%, 5% for the response categories). For simulating responses of *fixed pattern respondents*, we generated a repeating sequence of random numbers for each respondent (e.g., 1-2-3-1-2-3 . . . if the random numbers were 1 and 3).^3^ Modeling fixed response patterns with varying responses instead of repeating the same response across several consecutive items seems a more realistic setting than constantly choosing the same response option and it should disfavor detection via longstring or IRV. Sample sizes of simulated valid and careless respondents match those of the empirical study.

### Study 2: Empirical Study

#### Design and Participants

The web-based survey was administered through the survey tool *SoSci-Survey* (Leiner, 2020) between February 2020 and April 2020. Participants were recruited through social media, mailing lists, and a German online panel (Göritz et al., 2019). Participants were randomly assigned to one of two conditions in a between-subject design: regular responding or inattentive responding.^4^ The only difference between the two conditions was the instruction (see Appendix B in the OSF repository). The first instruction for regular response behavior was a standard instruction as given in many measures of typical behavior emphasizing that participants should select the answer that fits best after carefully considering each statement and taking the time to think about the answer. The second instruction tried to emulate the mind-set and response behavior of inattentive respondents (see also Niessen et al., 2016) by focusing on a speedy and superficial processing. Thus, the respondents were asked to quickly finish the survey without carefully reading the items. The instruction also emphasized the fact that participation in the lottery would not be affected by the actual responses. In addition to a data protection agreement, participants had to answer a control question (“What were your instructions?”) after reading the instruction and prior to the actual questionnaire to ensure that the instruction was read and understood. After the questionnaire, participants were asked whether they followed the instruction (yes/no) and had to answer the control question again.

Participants were included in the analyses if they had completed the questionnaire and indicated that they had followed and correctly remembered the instruction (for a flowchart with the dropout on the different stages see Figure S1 in the online repository). In total, 605 respondents completed the test, either under the regular condition (*n* = 361) or the careless responding condition (*n* = 244). Two thirds of the participants were female (66.9%, 32.4% male, and 0.7% diverse); the average age was 43.1 years (*SD* = 17.8). The sample of the regular and the careless condition was made up as follows: 28.1% pupils/students, 2.8% manual workers, 39.3% employees, 5.0% self-employed, 16.5% retired, and 8.3% others (information was available for *n* = 601). Almost all participants (>98%) stated that they understood German *well* or *very well* on a 4-point scale.

#### Selection of Analysis Sample

The empirical sample consisted of 605 respondents with 361 in the regular condition and 244 in the careless condition. For the statistical analyses, we split this sample into a test sample (30% or 180 respondents) and a training sample (70% or 425 respondents) with the following boundary condition: Based on the literature, we constrained the ratio of regular respondents to careless respondents in the test sample to 9:1 (or 162:18 in absolute numbers). The remaining respondents constituted the training sample with an almost balanced ratio (i.e., 199 regular and 226 careless respondents). A balanced ratio in the learning phase (i.e., training) and a realistic ration in the evaluation phase (i.e., test) provide optimal conditions, both from a learning and an evaluation perspective. The constrained random sampling procedure was repeated 1,000 times to quantify the variability of the results. The analysis sample derived from the empirical study corresponded to one of the conditions of the simulation study because both have a testing sample of 180 respondents, including 10% careless respondents (for more information see Appendix A in the OSF repository).

#### Measures

In addition to basic demographic data (i.e., age, gender, profession, command of the German language), participants worked on the German version of the HEXACO-60 (Ashton & Lee, 2009) measuring six personality traits: Honesty-Humility (H), Emotionality (E), Extraversion (X), Agreeableness (A), Conscientiousness (C), and Openness to Experience (O). Participants had to indicate their agreement to the different statements (e.g., “I would be quite bored by a visit to an art gallery”) on 5-point scales (1 = *strongly disagree*, 2 = *disagree*, 3 = *neutral*, 4 = *agree*, 5 = *strongly agree*). Skipping items was not possible to prevent test-takers from rushing through the questionnaire. We collected response times on the item level, which were winsorized for extremely long response times (i.e., replacing outliers with the 95th percentile) and aggregated to parcels of 10 (in accordance with the page layout) for subsequent analyses.

### Statistical Analyses for Studies 1 and 2

All traditional data-driven detection mechanisms (see also Table 1) were calculated with the R package careless (Version 1.1.3; Yentes & Wilhelm, 2018), except for the *Z*_h_ statistic that was calculated using mirt (Version 1.32.1; Chalmers, 2012). In more detail, the Mahalanobis distance was calculated (with a confidence level of 95%) on data sets in which reversely formulated items were recoded. Psychometric antonyms depict the within-person correlation of all negatively correlated item pairs, irrespective of the scale they belong to. A critical correlation between two items of *r* = −.20 was considered sufficient given the typical low mean item-intercorrelation of the HEXACO items of .05 [−.45; .63]. In contrast to semantic antonyms that take the item content into account, psychometric antonyms are purely data-driven (see also Curran, 2016). Even–odd consistency was computed as the within-person correlation between the even and the odd items of a subscale; that is, information on the six-dimensional structure of the HEXACO questionnaire was taken into account in the calculation of the coefficient. At least six identical responses in a row (before reverse coding negatively phrased items) were considered conspicuous for the longstring index (see also Niessen et al., 2016). IRV can manifest on both sides of the distribution, either as low IRV scores reflecting straight lined responses (Dunn et al., 2018) or as high IRV scores indicating highly random responses (Marjanovic et al., 2015). Accordingly, IRV scores were calculated twice and the higher accuracy rates were taken, which constitutes an overestimation, because in realistic settings such informed adjustments cannot be made. The *Z*_h_ person-fit statistic was based on a polytomous multidimensional item response theory (IRT) model with the HEXACO traits as dimensions (Drasgow et al., 1985).

We used the GBM algorithm of the R package gbm (Version 2.1.5; Greenwell et al., 2019) as supervised machine learning algorithm and caret (Kuhn, 2008, 2020) as interface for parallel modeling and prediction. To assess the correctness of the model classification, we split the sample into a holdout (also called test) sample (30%) to test model performance in an independent test sample and a training-validation sample (70%) to train and tune the model. To minimize overfitting (Yarkoni & Westfall, 2017), we used 10-fold cross-validation. The optimal settings for the tuning parameter for each of 1,000 random training-validation samples were identified via grid search with the following parameter settings: interaction depth of 2, 3, or 4, the minimum leaf size between 4 and 10, the shrinkage as a sequence between .001 and .03 in small steps of .002, and trees between 250 and 800 in steps of 50.

In the empirical study, the samples of the regular and careless respondents in the training sample were almost equal in size, providing optimal learning conditions for the algorithm. In the simulation study, however, we used the same prevalence of careless respondents in the data generation process for both the training and the test sample (5%, 10%, or 15% depending on the condition, see Appendix A for detailed information on the sample size). It is a well-known fact that machine learning algorithms that are trained on highly unbalanced data tend to bias the prediction in favor of the majority group leading to low sensitivity (e.g., Van Hulse et al., 2007). To avoid this statistical artifact, we used up-sampling (e.g., García et al., 2012), an often applied technique, in which observations in the minority group (i.e., careless respondents) are replicated to match the sample size of the majority group (i.e., regular respondents). Please note the test sample is unaffected by the up-sampling. The analyses were repeated 1,000 times for different random samples generated in the simulation study and randomly drawn samples in the empirical study (training vs. testing sample).

To evaluate the binary classification into careless respondents (CR) and regular respondents (RR), we report five performance metrics (for an overview see Tharwat, 2018) based on the number of correctly identified CR (true positives, TP), incorrectly identified CR (false positives, FP), correctly identified RR (true negatives, TN), and incorrectly identified RR (false negatives, FN): (a) sensitivity or true positive rate or recall (= TP/(TP + FN)), (b) specificity or true negative rate (= TN/(FP + TN)), (c) precision or positive predictive value (= TP/(TP + FP)), (d) accuracy (= (TP + TN)/(P + N)), and (e) the balanced accuracy, which is the mean of sensitivity and specificity. These indices were calculated in the test sample for each of the 1,000 iterations of the analyses.

## Results

### Study 1: Simulation Study

In the simulation study, we considered a 3 × 3 design, varying the amount of careless respondents in the overall sample (5%, 10%, 15%) and considering three types of response styles (random, midpoint, fixed pattern respondents). To reduce the complexity of the results, we only elaborate the results of the 10% condition because they were similar to the results of the other conditions (see Tables S1 and S2 in the online repository). Moreover, the size of the test sample (*n* = 180) in this condition is identical to the empirical data, which enables a comparison to the empirical data. Also, most frequently, prevalence rates between 10% and 15% are reported in the literature (Kurtz & Parrish, 2001; Meade & Craig, 2012). Table 2 summarizes the means and standard deviations for different performance metrics across 1,000 iterations for the three types of careless responding. The main findings of the simulation study are as follows: First, several detection methods performed well with respect to specificity; that is, regular respondents were in most cases correctly classified. In contrast, the rate of correctly identified careless respondents (i.e., sensitivity) is often low, which is in line with previous results (e.g., Niessen et al., 2016). Second, traditional detection mechanisms such as the Mahalanobis distance and the polytomous *Z*_h_ statistics performed excellent in terms of specificity and sensitivity for random respondents, whereas the sensitivity was low for the midpoint and pattern respondents. Third, although for the midpoint and pattern respondents the specificity for the traditional detection mechanisms (with the exception of the *Z*_h_ statistic) can be considered good, only the IRV yielded acceptable values for sensitivity. Fourth, the GBM performed excellent with balanced accuracies of .86 for random respondents, .94 for midpoint respondents, and .95 for pattern respondents. Also, the precision was high, indicating that only a small fraction of the flagged respondents were false positives (see Table 2). Please note that the high classification accuracy of the gradient boosted trees indicate that the up-sampling procedure mostly compensated for potential adverse effect of unbalanced groups in the training-validation sample. As the number of careless respondents in the training data set increases, the performance only slightly improves (see Tables S1 and S2 in the online repository for the 5% and 15% careless respondents condition).

**Table 2.** Classification Accuracy of Traditional and Machine Learning Algorithms With Simulated Data.

Item	Maha.	Antonyms	EvenOdd	Longstring	IRV	*Z*_h_	GBM
Random respondents							
Accuracy	1.00 (.00)	.87 (.02)	.88 (.02)	.83 (.02)	.96 (.01)	.92 (.02)	.96 (.01)
Sensitivity	1.00 (.01)	.11 (.07)	.49 (.12)	.01 (.03)	.80 (.07)	1.00 (.00)	.74 (.11)
Specificity	1.00 (.00)	.96 (.02)	.93 (.02)	.93 (.02)	.98 (.01)	.91 (.02)	.99 (.01)
Precision	.97 (.04)	.22 (.15)	.43 (.09)	.02 (.04)	.80 (.07)	.55 (.05)	.90 (.08)
Balanced accuracy	1.00 (.01)	.53 (.04)	.71 (.06)	.47 (.02)	.89 (.04)	.95 (.01)	.86 (.05)
Midpoint respondents							
Accuracy	.91 (.01)	.90 (.02)	.88 (.02)	.87 (.02)	.94 (.02)	.74 (.03)	.98 (.01)
Sensitivity	.18 (.08)	.27 (.10)	.50 (.12)	.37 (.11)	.68 (.09)	.51 (.12)	.88 (.08)
Specificity	.99 (.01)	.97 (.02)	.93 (.02)	.93 (.02)	.96 (.01)	.77 (.02)	.99 (.01)
Precision	.65 (.22)	.48 (.16)	.43 (.09)	.36 (.10)	.68 (.09)	.20 (.04)	.90 (.07)
Balanced accuracy	.59 (.04)	.62 (.05)	.71 (.06)	.65 (.06)	.82 (.05)	.64 (.06)	.94 (.04)
Pattern respondents							
Accuracy	.87 (.01)	.80 (.06)	.86 (.02)	.85 (.02)	.95 (.02)	.72 (.03)	.96 (.02)
Sensitivity	.03 (.04)	.08 (.13)	.10 (.08)	.20 (.09)	.76 (.10)	.50 (.11)	.93 (.08)
Specificity	.96 (.01)	.88 (.06)	.93 (.02)	.93 (.02)	.97 (.01)	.75 (.03)	.96 (.02)
Precision	.09 (.11)	.06 (.10)	.11 (.08)	.23 (.10)	.76 (.10)	.18 (.04)	.74 (.10)
Balanced accuracy	.50 (.02)	.48 (.07)	.51 (.04)	.56 (.05)	.87 (.06)	.63 (.06)	.95 (.04)

*Note*. Results are means and standard deviations across 1,000 simulated data sets (*n*_test_ = 180 with 10% careless respondents). Maha. = Mahalanobis distance; Antonyms = psychometric antonyms; EvenOdd = even–odd consistency; Longstring = Longstring Index; IRV = intraindividual response variability; *Z*_h_ = polytomous IRT person-fit statistic; GBM = gradient boosting machine. IRT = item response theory.

### Study 2: Empirical Study

To compare the detection mechanisms for the empirical study, we first present the different performance metrics (see Table 3). To illustrate the variability of the empirical results, we drew 1,000 test samples from the empirical data (i.e., 30% of the overall sample) that comprises 90% regular and 10% careless respondents (i.e., 162 and 18 respondents, respectively, in absolute numbers) and report means and standard deviations of the respective classification indices. In addition to a GBM that relied on the response vectors (GBM_Res_), we included two models that used response times only (GBM_RT_) and a combination of both information (GBM_Res+RT_), respectively. Since the sample size of the holdout sample is identical to the setup of the simulation study, the results can be compared with the simulation results reported above. In comparison to the simulated conditions, all detection mechanisms performed worse in the empirical study, demonstrating that it is much harder to flag aberrant responses. For the traditional detection methods, the picture that has already emerged for the midpoint and the pattern respondents in the simulation study was replicated with good values for specificity, but low values for sensitivity. Importantly, sensitivity and specificity were only mediocre for the machine learning approach: The average *false positive rate* (= 1 − specificity) was .29 for the most complex model and the average *miss rate* (or false negative rate = 1 − sensitivity) was .40. The *precision* (or positive predictive value) as the proportion of correctly identified careless respondents out of all flagged respondents is only .19, which means that the flagging of participants is false in 81% of the cases.

**Table 3.** Classification Accuracy of Traditional and Machine Learning Algorithms With Empirical Data (10% Prevalence).

Item	Maha.	Antonyms	EvenOdd	Longstring	IRV	*Z*_h_	GBM_Res_	GBM_RT_	GBM_Res + RT_
Accuracy	.80 (.02)	.86 (.02)	.84 (.02)	.82 (.02)	.83 (.01)	.67 (.02)	.59 (.04)	.70 (.05)	.70 (.04)
Sensitivity	.18 (.08)	.11 (.08)	.19 (.09)	.18 (.09)	.14 (.07)	.38 (.11)	.61 (.12)	.56 (.12)	.60 (.12)
Specificity	.87 (.02)	.94 (.02)	.91 (.02)	.90 (.02)	.90 (.01)	.70 (.02)	.58 (.04)	.71 (.05)	.71 (.05)
Precision	.13 (.06)	.17 (.11)	.19 (.08)	.16 (.07)	.14 (.07)	.12 (.03)	.14 (.02)	.18 (.04)	.19 (.04)
Balanced accuracy	.53 (.04)	.53 (.04)	.55 (.04)	.54 (.04)	.52 (.04)	.54 (.06)	.60 (.06)	.64 (.06)	.66 (.06)

*Note*. Results are means and standard deviations across 1,000 random test samples of the empirical data (*n*_test_ = 180 with 10% careless respondents). Maha. = Mahalanobis distance; Antonyms = psychometric antonyms; EvenOdd = even–odd consistency; Longstring = Longstring Index; IRV = intraindividual response variability; *Z*_h_ = polytomous IRT person-fit statistic; GBM = gradient boosting machine; Res = responses; RT = response times. IRT = item response theory.

*Sensitivity*, as the proportion of careless respondents that are correctly identified, and *specificity*, which denotes the proportion of regular respondents that are correctly identified, are mutually dependent—the increase of one leads to a reduction of the other (i.e., sensitivity-specificity trade-off). In the present context, we used an optimization function for the GBM algorithm that took into account both performance metrics equally. Depending on the research question and the consequences of misclassification other priorities might be better suited. Thus, researchers should ask whether Type I and Type II errors are equally unfavorable for their research or application, a question that can only be answered depending on the consequences of incorrect classifications. Improperly flagging responses as careless (Type I error) may unduly reduce the sample size, increase the survey costs, and bias the results, while not detecting real careless respondents (Type II error) might also bias and invalidate test results (also known as the MTurk quality crisis, e.g., Kennedy et al., 2020). In our analysis, we used a negative logistic loss function for the gradient boost algorithm to make the model more sensitive to the class of careless respondents, resulting in higher balanced accuracy rates (i.e., arithmetic mean of sensitivity and specificity) in comparison to the traditional detection mechanisms. Adopting a more conservative approach, that is, raising the bar to flag potential careless respondents, the accuracy or the receiver operating characteristic curve metric would be a better metric to optimize.

**Figure 2.**
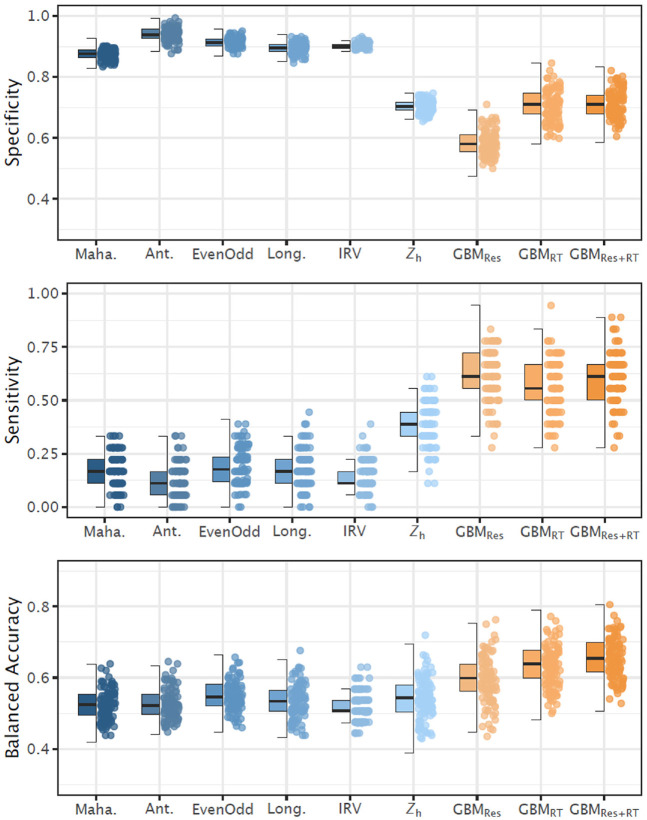
Specificity, sensitivity, and balanced accuracy across detection mechanisms. Maha. = Mahalanobis distance; Ant. = psychometric antonyms; EvenOdd = even–odd consistency; Long. = Longstring Index; IRV = intraindividual response variability; *Z*_h_ = polytomous IRT person-fit statistic; GBM = gradient boosting machine; Res = responses; RT = response times. Left side: The boxplot reflects the interquartile range, the solid line represents the median, and the whiskers represent minimum/maximum values within 1.5 times the interquartile range. Right side: Jittered point plot of 100 randomly drawn values. IRT = item response theory.

In addition to the *sensitivity* and *specificity*, we present the *balanced accuracy*—that is, the arithmetic mean of sensitivity and specificity—as a compromise between both performance measures of a binary classification test (see Figure 2). The main findings concerning balanced accuracy can be summarized as follows. First, both the traditional detection mechanisms and the machine learning algorithm performed considerably worse in the realistic setting versus the simulated data condition. This is particularly evident in the *precision*, which in the empirical study reached only about one fourth of the values from the simulation study. Second, the GBM achieved a slightly better trade-off between sensitivity and specificity. The balanced accuracy was best if the responses and the (aggregate) response times were included in the model. It should be mentioned that adding further predictors such as the number of response option changes, or the traditional detection measures did not lead to a substantial increment in the prediction quality in our study. The values had a large variation; that is, the performance metrics varied considerably depending on the specific sample. This is most likely due to the small sample size (for machine learning standards). It can also be taken as evidence that careless response processes under real-world conditions are much more heterogeneous than artificial ones (see also Denison & Wiernik, 2020) and, therefore, require a larger learning pool.

## Discussion

Comparing the results of the simulation and the empirical study showed substantial differences in almost all performance measures for the detection of careless respondents (cf., Hong et al., 2020; Meade & Craig, 2012; Niessen et al., 2016). Whereas the simulation study highlighted substantial advantages of the studied machine learning algorithm, respective benefits were more modest in the empirical application. One reason for this discrepancy might be that some “regular respondents” in the empirical study might have (at least in part) responded carelessly, even though they claimed to adhere to the instructions. In turn, there also might have been “careless respondents” that actually paid attention out of curiosity, reactance, or misunderstanding. Both effects most likely restricted the maximum obtainable sensitivity in the empirical study. Thus, the best specificity value found was .71 for the GBM, which might constitute an upper bound that can be expected in applied settings. Another explanation for the observed discrepancy between the two studies might be that the simulated types of careless responding are inferior characterizations of actual careless responding observed in the real world. For example, humans are usually not capable of generating completely random answers as assumed in many previous simulation studies; a circumstance that is incidentally also exploited in the detection of malingering (Orthey et al., 2019). Apparently, human’s response behavior is much more erratic than what is assumed in rather straightforward, pattern-like responses of simulation studies. A somewhat disappointing conclusion, therefore, is that findings of simulation studies do not directly translate into an ecologically valid context.

The present study falls in line with a series of critical voices on the hype about machine learning algorithms in fields such as personality science (e.g., Stachl et al., 2020) or clinical research (Cearns et al., 2019). In psychology, data quality and structure are frequently not comparable to computer sciences. We often rely on small sample sizes, which require special attention to avoid overfitting (Vabalas et al., 2019). Moreover, typical indicators in psychological research such as questionnaire or test data are subject to measurement errors, which might obscure the true relationship underlying the data (Jacobucci & Grimm, 2020). Careless (and also regular) responding might also manifest in many idiosyncratic ways that simply do not follow a clear predictable pattern. All these circumstances might account for the sobering results regarding the usefulness of machine learning in psychology.

### The Potential of Machine Learning Algorithms

Gradient boosted trees are effective in cases where patterns in relation to a labeled outcome can be derived. In the simulation study, the GBM could “learn” not only because the pattern of careless (and regular) responding was rule-based and separable from each other but also because the outcome was provided. This knowledge of the outcome is used to optimally set up the predictive model. In contrast, traditional detection mechanisms are unsupervised algorithms that operate without such information, which makes it an almost unfair comparison. However, in real settings, information on the extent of regular responding is not given and one has to rely on other (partially unreliable) information such as asking participants if they answered diligently. One advantage of the GBM is that they can easily deal with plenty of features and split them optimally. In case of response times, GBM can flexibly handle this additional source of information, whereas traditional detection mechanisms have to work with a predefined cutoff. The importance of response times in the context of detecting aberrant responses has also been stressed elsewhere (e.g., Leiner, 2019). Usually, detection mechanisms based on response times focus on extremely rapid responses (e.g., 2 seconds/item; Huang et al., 2012; Wise & Kong, 2005). However, a speedy response is not necessarily synonymous with careless responding. In turn—although the instruction of the careless responding condition specifically requested to get through the questionnaire “as quickly as possible”—it is also conceivable that a respondent is unusually slow (see also Niessen et al., 2016), for example, if they are distracted. In principle, the GBM algorithm can detect either very fast or extremely slow respondents. In the present study, however, we observed a huge overlap of the response time distributions across conditions and larger differences at the lower tail of the distribution (see Figure S2 in the online repository).

Arguably, there is still potential to improve the classification accuracy of the GBM since even the “best” model is not optimal for practical purposes. For example, the predication model could incorporate additional information such as scales measuring test-taking motivation, conscientiousness, or diligence as additional predictors. It might also be worthwhile to use bogus items, instructed response items, or even paradata that are much harder to fake (e.g., Buchanan & Scofield, 2018; Steger, Schroeders, & Wilhelm, 2020). One promising candidate for such a piece of information would be the item number indicating the progress in the completion of the questionnaire. Careless responding is not dichotomous, rather insufficient effort can take place sooner or later depending on the commitment or fatigue of the test-taker and the length of the questionnaire (Gibson & Bowling, 2020). Costs (e.g., time or cognitive effort) and benefits (e.g., financial gratification or intrinsic motivation) are set off against each other to evaluate the willingness to further participate at a specific point of time. That is, not only the willingness to answer superficially increases but also the probability to abort the questionnaire (Bowling et al., 2020). Thus, it seems plausible to consider the phenomena of attrition and careless responding in tandem (Meade et al., 2017). Yet even in a complete data set, items at the beginning of the questionnaire might be more decisive for distinguishing careless responding than those items at the end, because it is likely that the motivation of even engaged participants decreases till the end of the questionnaire (see also Wise & Kong, 2005).

### Disadvantages of Available Detection Indices

A serious drawback of several traditional detection indices (e.g., Longstring Index, psychometric antonyms) is that the cutoffs are usually set manually and data-driven without controlling for the risk of overfitting, which is why recommendations for cutoffs vary in the literature depending on the data set used to derive them (e.g., Niessen et al., 2016). Nonetheless, traditional indicators to detect careless responding—whether statistical outlier methods, consistency analyses, and response pattern functions—have the advantage that they can be applied with minimal prerequisites to new questionnaires. In contrast, supervised machine learning algorithms such as gradient boosted trees require labeled data sets, need an extensive training phase, and the prediction models are bound to a specific set of items. Generalizations to other data sets, samples, and situations are *not* possible, because every examination is highly specific in terms of items and persons. These are major drawbacks. In principle, it would be possible to follow the design we outlined in the study—that is, implement an experimental prestudy to build a “detection model” and to subsequently clean the data of the main study. However, given the present set of results, we are skeptical that such an approach will provide satisfying results because careless responding in real life is apparently a lot harder to detect than obvious misconduct.

We would also like to caution that decisions of traditional detection approaches, which test-takers respond carelessly, are based not only on the gathered data but also on theoretical assumptions of the data generation process itself. Model-based approaches rely on the assumption that normality can be captured sufficiently well by a given model, so that non-matching observations are considered outliers. For example, in the even–odd consistency analyses, participants whose answers to similar questions are contradictory or inconsistent are more conspicuous. However, such a categorization involves certain risks, since it decides a priori on the correctness of data, and flags data that are not in line with a certain model or deviate the most. However, these model assumptions can be erroneous themselves, bearing the risk of shaping the data until it fits the model assumption, which might render the cure worse than the disease. Also, using machine learning algorithms comes at a cost because they are more data-driven and are considered black box approaches. As an outlook for future research, it seems promising to reunite the approaches of explanation and prediction (Yarkoni & Westfall, 2017) by enriching machine learning with assumptions about the process (and ultimately the causes) of careless responding (e.g., the risk to engage in careless responding should increase with test length and cognitive demand, which could be accounted for in the modeling procedure).

## Supplemental Material

sj-pdf-1-epm-10.1177_00131644211004708 – Supplemental material for Detecting Careless Responding in Survey Data Using Stochastic Gradient Boosting Click here for additional data file. Supplemental material, sj-pdf-1-epm-10.1177_00131644211004708 for Detecting Careless Responding in Survey Data Using Stochastic Gradient Boosting by Ulrich Schroeders, Christoph Schmidt and Timo Gnambs in Educational and Psychological Measurement

## Notes

1 The differences between the detection measures that belong to the group of consistency analysis versus the response pattern functions, thus, is the point of comparison: In the former, it is mainly on the individual response vector, while in the latter, it also takes into account the responses of the sample. Please note that the distinction is not clear-cut and some mechanisms can be assigned differently (e.g., psychometric antonyms).

2 The random respondents correspond to the condition of “uniformly random careless data” in Meade and Craig (2012), whereas midpoint respondents correspond to the condition of “normally distributed random careless data.” The fixed pattern respondents were not previously simulated.

3 We used the R code rep(sample.int(5,1):sample.int(5,1), length.out=60) to generate the sequence.

4 The design also included a third condition that instructed respondents to deliberately respond carelessly with the intention not to be discovered. This induced response style resembles faking behavior (Geiger et al., 2018), but without impression management. Instead, test-takers were instructed to complete the questionnaire quickly without being recognized as an inattentive respondent. To streamline the present article, this condition has not been examined further.
