# Supplementary material for: Detecting Careless Responding in Survey Data Using Stochastic Gradient Boosting
Source: Educ Psychol Meas. 2021 Apr 19;82(1):29–56. doi: 10.1177/00131644211004708 (PMC8725053; doi:10.1177/00131644211004708)
Supplement: sj-pdf-1-epm-10.1177_00131644211004708 – Supplemental material for Detecting Careless Responding in Survey Data Using Stochastic Gradient Boosting [file sj-pdf-1-epm-10.1177_00131644211004708.pdf]

Appendix A

Simulation Study (n = 600)

Prevalence CR

5%[a]

	RR	CR	Σ
Test (30%)	171	9	180
Training (70%)	369	51	420
Σ	540	60	600

10%[b]

	RR	CR	Σ
Test (30%)	162	18	180
Training (70%)	378	42	420
Σ	540	60	600

15%[c]

	RR	CR	Σ
Test (30%)	153	27	180
Training (70%)	387	33	420
Σ	540	60	600

Empirical Study (n = 605)

Prevalence CR

10%[b]

	RR	CR	Σ
Test (30%)	162	18	180
Training (70%)	199	226	425
Σ	361	244	605

Note. RR = Regular Respondents; CR = Careless Respondents. [a] Results are reported in Table OS 1 (online supplement); [b] Results are reported in the manuscript; [c] Results are reported in Table OS 2.

Appendix B

Instruction regular responding

On the following pages you will find a series of statements about you. Please read each statement and decide how much you agree or disagree with that statement. Then write your response in the space next to the statement using the following scale [...]. Please answer every statement, even if you are not completely sure of your response. Please take your time to think about each answer carefully.

Instruction inattentive responder

Imagine that you have to complete a questionnaire in order to take part in the lottery. You do not feel like completing the questionnaire. You are only interested in getting through the questionnaire as quickly as possible. Irrespective of your answers, you will take part in the lottery if you finish the entire questionnaire. Please complete the following questionnaire like you would have done if you were in this situation.

Instruction cheating responder

Imagine that you have to complete a questionnaire in order to take part in the lottery. You do not feel like completing the questionnaire. You are only interested in quickly getting through the questionnaire without being directly spotted as superficial responder. Irrespective of your answers, you will take part in the lottery if you finish the entire questionnaire. Please complete the following questionnaire like you would have done if you were in this situation.

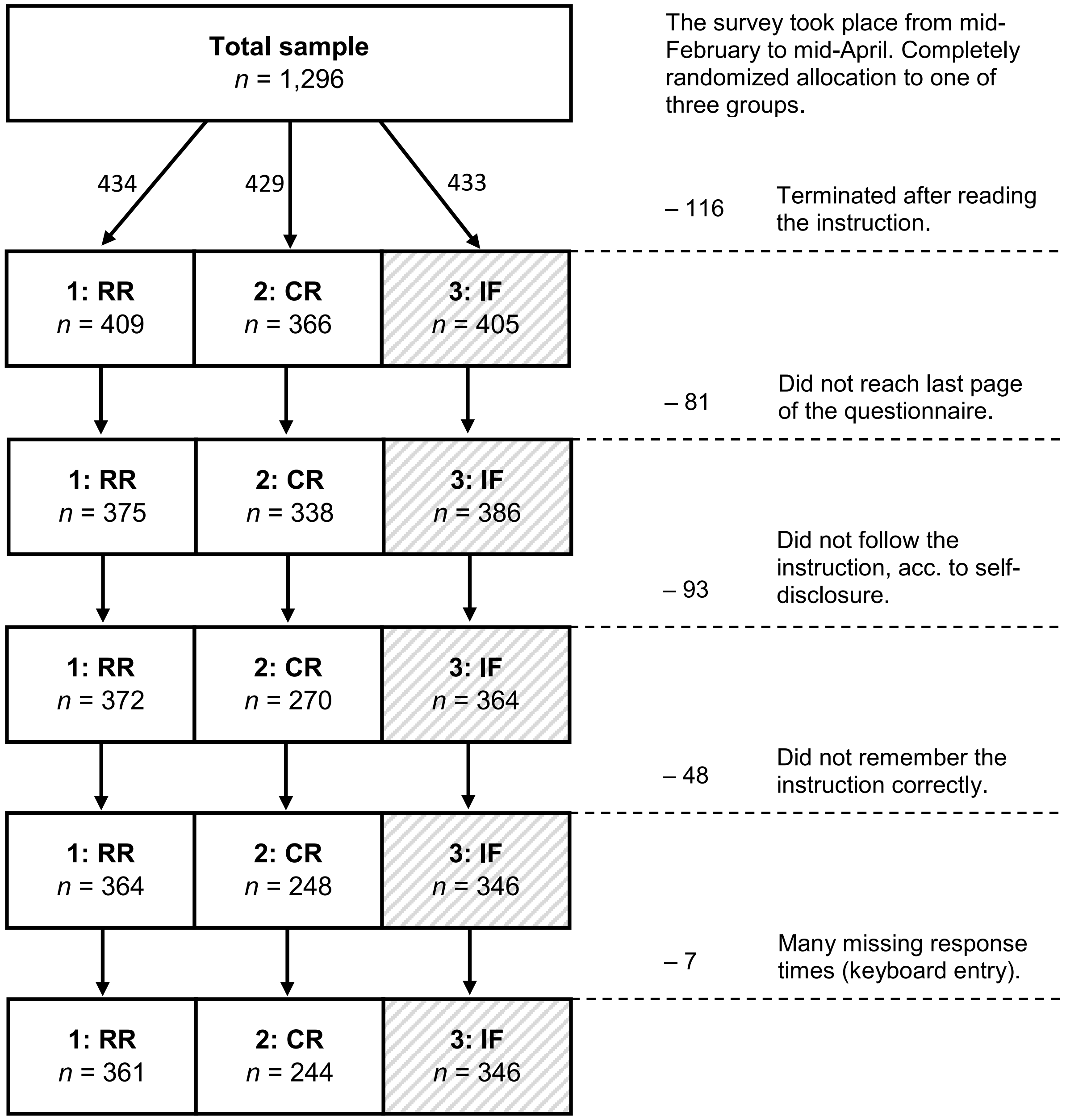

Figure OS 1. Sample Size Attrition Across Condition. RR = Regular Respondents; CR = Careless Respondents; IF = Instructed Faker. Please note that in online panel studies participant usually only get paid or receive some kind of gratification if they work accurately and diligently. Doing the opposite apparently confused some participants, others self-disclosed that they did not adhere to the instructions, which lead to a higher dropout rate in the careless responding condition.

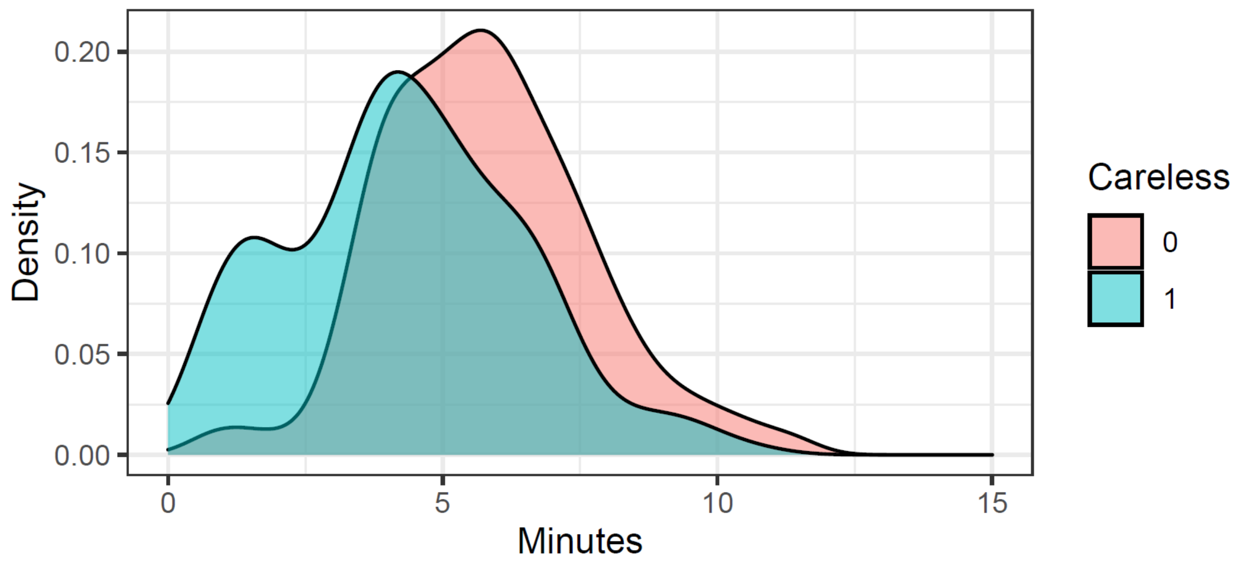

Figure OS 2: Duration of the Test Across Respondents Groups. For the calculation we winsorized the response times per item to exclude extremely large individual response times.

Table OS 1. Classification Accuracy of Traditional and Machine Learning Algorithms With Simulated Data (5% prevalence)

	Maha.	Antonyms	EvenOdd	Longstring	IRV	Z_h	GBM
Random Respondents							
Accuracy	.99 (.01)	.92 (.02)	.90 (.02)	.88 (.02)	.97 (.01)	.85 (.02)	.98 (.01)
Sensitivity	1.00 (.01)	.10 (.10)	.50 (.17)	.01 (.04)	.71 (.12)	1.00 (.00)	.79 (.14)
Specificity	.99 (.01)	.96 (.02)	.93 (.02)	.93 (.02)	.98 (.01)	.84 (.02)	.99 (.01)
Precision	.88 (.09)	.12 (.14)	.27 (.09)	.01 (.03)	.71 (.12)	.25 (.03)	.82 (.13)
Balanced Accuracy	1.00 (.01)	.53 (.05)	.71 (.08)	.47 (.02)	.85 (.06)	.92 (.01)	.89 (.07)
Midpoint Respondents							
Accuracy	.95 (.01)	.93 (.02)	.90 (.02)	.90 (.02)	.96 (.01)	.75 (.02)	.98 (.01)
Sensitivity	.25 (.14)	.25 (.15)	.50 (.17)	.38 (.16)	.58 (.13)	.64 (.17)	.91 (.09)
Specificity	.99 (.01)	.96 (.02)	.93 (.02)	.93 (.02)	.98 (.01)	.76 (.02)	.99 (.01)
Precision	.52 (.25)	.28 (.17)	.27 (.09)	.21 (.09)	.58 (.13)	.12 (.03)	.80 (.12)
Balanced Accuracy	.62 (.07)	.61 (.07)	.71 (.09)	.65 (.08)	.78 (.07)	.70 (.09)	.95 (.05)
Pattern Respondents							
Accuracy	.94 (.01)	.88 (.04)	.89 (.02)	.89 (.02)	.98 (.01)	.74 (.03)	1.00 (.01)
Sensitivity	.10 (.09)	.10 (.15)	.10 (.11)	.19 (.13)	.76 (.14)	.56 (.16)	.97 (.07)
Specificity	.98 (.01)	.92 (.04)	.93 (.02)	.93 (.02)	.99 (.01)	.75 (.03)	1.00 (.00)
Precision	.22 (.21)	.07 (.10)	.05 (.06)	.12 (.08)	.75 (.14)	.11 (.03)	.95 (.07)
Balanced Accuracy	.54 (.04)	.51 (.07)	.51 (.06)	.56 (.07)	.87 (.07)	.66 (.08)	.98 (.03)

Note. Results are means and standard deviations across 1,000 simulated data sets (n_{test} = 180 with 5% careless respondents). Maha. = Mahalanobis Distance; Antonyms = Psychometric antonyms; EvenOdd = Even-odd-consistency; Longstring = Longstring Index; IRV = Intraindividual response variability; Z_h = Polytomous IRT person fit statistic; GBM = Gradient Boosting Machine.

Table OS 2. Classification Accuracy of Traditional and Machine Learning Algorithms With Simulated Data (15% prevalence)

	Maha.	Antonyms	EvenOdd	Longstring	IRV	Z_h	GBM
Random Respondents							
Accuracy	1.00 (.00)	.83 (.02)	.86 (.02)	.79 (.02)	.95 (.02)	.96 (.01)	.94 (.02)
Sensitivity	.99 (.02)	.12 (.06)	.50 (.10)	.01 (.02)	.84 (.05)	1.00 (.00)	.67 (.10)
Specificity	1.00 (.00)	.95 (.02)	.93 (.02)	.93 (.02)	.97 (.01)	.95 (.02)	.99 (.01)
Precision	.99 (.02)	.31 (.16)	.55 (.09)	.03 (.05)	.84 (.05)	.78 (.06)	.94 (.06)
Balanced Accuracy	.99 (.01)	.53 (.03)	.71 (.05)	.47 (.02)	.90 (.03)	.97 (.01)	.83 (.05)
Midpoint Respondents							
Accuracy	.86 (.01)	.86 (.02)	.86 (.02)	.84 (.02)	.92 (.02)	.71 (.03)	.97 (.01)
Sensitivity	.14 (.07)	.27 (.08)	.50 (.09)	.37 (.09)	.73 (.06)	.41 (.09)	.84 (.07)
Specificity	.99 (.01)	.97 (.02)	.93 (.02)	.93 (.02)	.95 (.01)	.77 (.03)	.99 (.01)
Precision	.70 (.22)	.60 (.15)	.55 (.09)	.47 (.10)	.73 (.06)	.24 (.05)	.94 (.05)
Balanced Accuracy	.56 (.03)	.62 (.04)	.71 (.05)	.65 (.05)	.84 (.04)	.59 (.05)	.92 (.04)
Pattern Respondents							
Accuracy	.79 (.02)	.75 (.07)	.82 (.02)	.82 (.02)	.93 (.02)	.69 (.04)	.98 (.01)
Sensitivity	.01 (.02)	.05 (.10)	.10 (.07)	.20 (.08)	.76 (.08)	.45 (.09)	.91 (.08)
Specificity	.92 (.02)	.87 (.08)	.93 (.02)	.93 (.02)	.96 (.01)	.73 (.04)	1.00 (.00)
Precision	.03 (.04)	.05 (.10)	.16 (.10)	.32 (.11)	.76 (.08)	.23 (.05)	.98 (.03)
Balanced Accuracy	.47 (.01)	.46 (.06)	.51 (.03)	.56 (.04)	.86 (.05)	.59 (.06)	.95 (.04)

Note. Results are means and standard deviations across 1,000 simulated data sets (n_{test} = 180 with 15% careless respondents). Maha. = Mahalanobis Distance; Antonyms = Psychometric antonyms; EvenOdd = Even-odd-consistency; Longstring = Longstring Index; IRV = Intraindividual response variability; Z_h = Polytomous IRT person fit statistic; GBM = Gradient Boosting Machine.
